# Physical activity and the Neuro-Immuno-Fascial interface in cancer patients: mechanisms, clinical evidence, and therapeutic integration

**DOI:** 10.3389/fonc.2026.1894580

**Published:** 2026-07-31

**Authors:** Stephanie Otto, An Thuy Ngo-Huang, Werner Klingler

**Affiliations:** 1 Comprehensive Cancer Center Ulm (CCCU), Ulm University Hospital, Ulm, Germany; 2 Department of Gynaecology and Obstetrics, Ulm University Hospital, Ulm, Germany; 3 Department of Internal Medicine I, Ulm University Hospital, Ulm, Germany; 4 Department of Internal Medicine III, Ulm University Hospital, Ulm, Germany; 5 Fascia Research Society (FRS), Burnsville, MN, United States; 6 Department of Palliative, Rehabilitation, and Integrative Medicine, The University of Texas MD Anderson Cancer Center, Houston, TX, United States; 7 Fascia Research Group, Department of Neurosurgery, Ulm University Hospital, Ulm, Germany; 8 Professorship for Conservative and Rehabilitative Orthopaedics, TUM School of Medicine and Health, Technical University Munich, Munich, Germany; 9 Faculty of Health, Queensland University of Technology, Brisbane, QLD, Australia; 10 Department of Anaesthesiology, Stiftung Rehabilitation Heidelberg (SRH) Hospital Sigmaringen, Sigmaringen, Germany

**Keywords:** exercise oncology, neuro-immuno-fascial interface, fascia mechanobiology, tumor microenvironment remodeling, heart rate variability, cancer-associated fibroblasts, immune checkpoint inhibitor synergy, cancer survivorship

## Abstract

Physical activity has progressed from supportive care to an evidence-based oncologic intervention, with large cohort studies showing dose-dependent reductions in all-cause cancer mortality of roughly 20–47% across multiple tumor entities, and randomized trial evidence supporting a pooled reduction of approximately 26%. The CCTG CO.21 CHALLENGE trial, the first randomized controlled trial powered for survival endpoints, demonstrated that structured post-adjuvant exercise reduced disease recurrence by 28% and mortality by 37% in stage II–III colon cancer survivors, with an effect size comparable to adjuvant chemotherapy. This narrative review proposes the Neuro-Immuno-Fascial (NIF) interface as a hypothetical integrative axis that may help explain how exercise influences tumor-related biology. Clinical trials have established that physical activity improves function, mitigates fatigue, and lowers recurrence risk in selected cohorts; however, the multi-step causal chain linking mechanical fascial remodeling to anti-tumor outcomes in humans remains speculative. Accordingly, the NIF construct is presented as a conceptual framework for translational research rather than a clinical decision-making tool. At the fascial–stromal interface, preclinical data suggest that exercise-induced mechanical loading may activate Piezo1/YAP–TGF β1 mechanotransduction cascades in cancer-associated fibroblasts and modulate immunosuppressive architecture via three convergent, yet exploratory, mechanisms: viscoelastic and hydration-driven remodeling of the extracellular matrix that may improve compliance and interstitial fluid dynamics; Piezo channel–mediated mechanosensing that may influence myokine release (e.g., interleukin 6, irisin, SPARC) and associate with downstream changes in fibroblast behavior and natural killer cell trafficking; and autonomic rebalancing, reflected in 20–50% improvements in heart rate variability, which may attenuate pro-tumorigenic sympathetic tone. Direct human evidence that training-induced changes in fascial dynamics causally drive anti-tumor immunity is currently lacking. Preclinical findings further indicate that exercise-induced vascular normalization can increase tumor vessel perfusion and reduce hypoxia, although the magnitude and clinical relevance of these shifts in patients require further validation. On this basis, we outline three priorities for translational exercise oncology: standardized fascial and autonomic phenotyping using shear-wave elastography and heart rate variability spectral analysis; tumor microenvironment phenotype–specific exercise dosing algorithms; and prospective trials combining aerobic and resistance training with myofascial interventions and immune checkpoint inhibition. Structured physical activity should remain a standard component of oncologic care, while NIF-informed phenotyping and dosing strategies warrant systematic investigation.

## 1 Introduction

**Figure 1:**
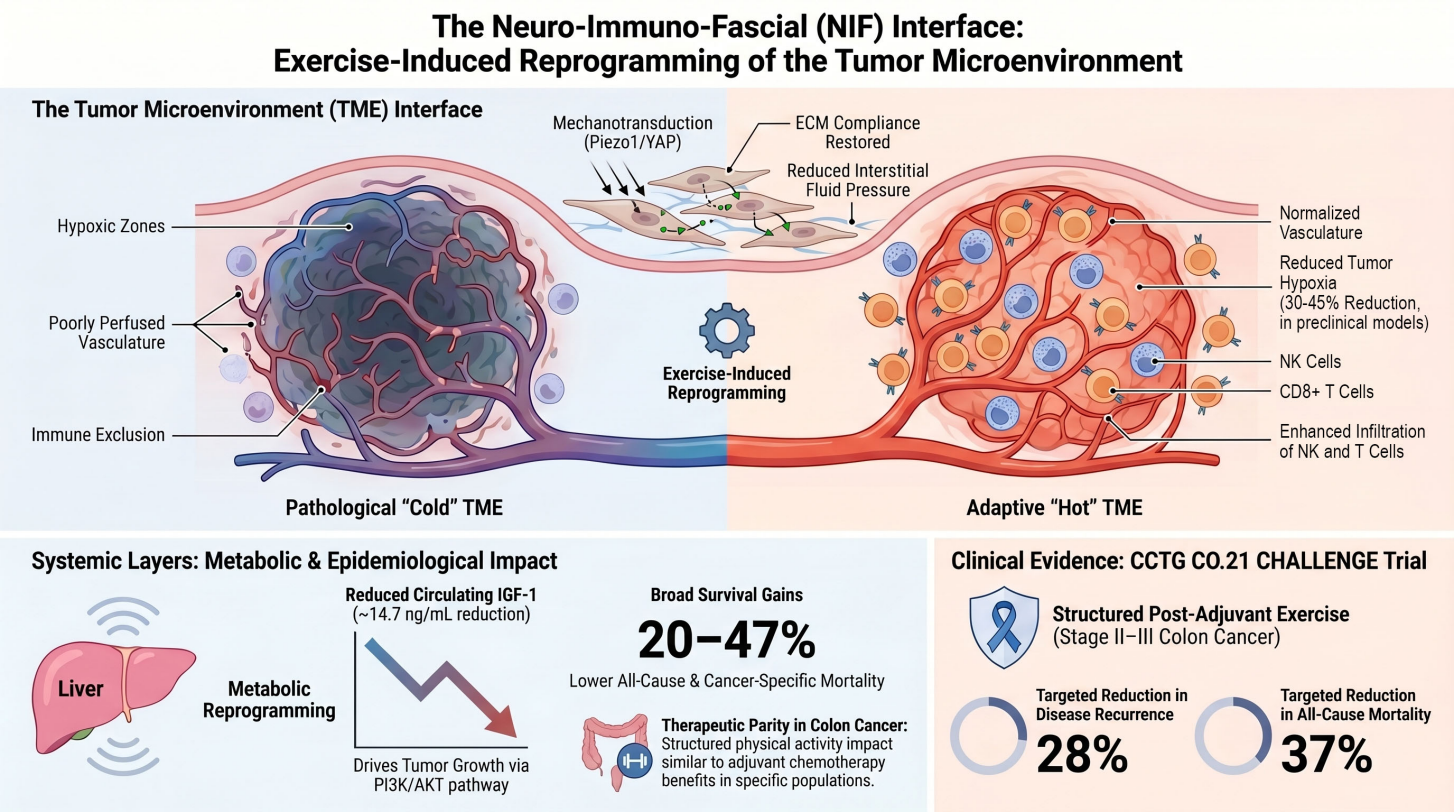
Graphical abstract. The Neuro-Immuno-Fascial (NIF) Interface: a conceptual model of exercise-associated tumor microenvironment adaptation. The Tumor Microenvironment (TME) Interface (Top): Schematic comparison between a pathological “cold” TME (left) and a putative exercise-associated adaptive “hot” TME (right). The pathological “cold” TME is characterized by hypoxic zones, poorly perfused vasculature, and immune exclusion. Preclinical models suggest that physical activity may influence mechanotransduction pathways (e.g., Piezo1/YAP signaling within the fascial matrix), with potential downstream effects on extracellular matrix (ECM) compliance, interstitial fluid pressure, vascular function, and immune-cell infiltration. The depicted 30–45% reduction in tumor hypoxia is derived from preclinical models and should not be interpreted as an established average effect in human oncology populations. Systemic Layers: Metabolic and Epidemiological Impact (Bottom Left): Exercise is associated with systemic metabolic adaptations, including reductions in circulating IGF-1 in some cancer survivor cohorts, alongside epidemiological associations with lower all-cause and cancer-specific mortality. These systemic observations provide clinical context for the conceptual model but do not establish a direct fascial-mediated anti-tumor mechanism in humans. Clinical Evidence (Bottom Right): Summary of the CCTG CO.21 CHALLENGE trial in stage II–III colon cancer survivors, showing reduced disease recurrence and all-cause mortality with structured post-adjuvant exercise. These outcomes support exercise as an evidence-based oncologic intervention in specific populations, while the NIF interface remains hypothesis-generating.

Cancer incidence and mortality remain leading global health challenges, with ~20 million new cases and 10 million deaths annually (1). Contemporary cancer therapeutics - chemotherapy, radiation, surgical dissection, targeted agents, immunotherapy - improve survival but cause toxicities compromising quality of life and adherence, including chemotherapy-induced peripheral neuropathy (CIPN), pain, cardiotoxicity, cognitive impairment, fatigue, lymphedema, dyspnea, physical deficits, and psychological morbidity (2, 3). Cancer survivors also face metabolic complications like obesity, diabetes, and cardiovascular disease (4). Structured physical activity (PA) has emerged as a therapeutic modality with efficacy comparable to pharmacological agents for symptom management and functional recovery (5). The evidence base has evolved from observational associations to randomized trials showing direct cancer outcomes influence; landmark data from the CHALLENGE trial (n=889 stage II-III colon cancer survivors) demonstrated a 3-year supervised exercise program reduced recurrence/new primaries by 28% and mortality by 37% (median follow-up 7.9-8.9 years post-randomization) (6). Crucially, because these robust survival data are restricted to stage II–III colon cancer survivors, they must not be generalized as an established “cancer-modifying” effect across all heterogeneous tumor types, treatment phases, or patient populations (6). PA modalities in oncology span endurance, resistance (for strength), and coordinative training; emerging strategies target fascial tissue - a dense, collagenous, mechanosensitive network altered in the tumor microenvironment (7). This review synthesizes the established clinical benefits of physical activity alongside these emerging, speculative mechanobiological pathways, specifically exploring the proposed, non-validated interactions within the fascia-autonomic nervous system axis (**Figure 1**). The evidence base draws on structured searches of PubMed, the Cochrane Database, and Web of Science (2006–2026), prioritizing RCTs, systematic reviews, and meta-analyses assessed per GRADE criteria; full methodological details and a transparent matrix of all 80 included studies (**Supplementary Table 1**) are provided in the following section.

## 2 Methodology and evidence synthesis

A structured literature review was conducted to synthesize clinical, translational, and preclinical evidence, as detailed below.

### 2.1 Search strategy and study selection

This narrative review was based on a structured search of English-language publications from 2006 to January 2026 in PubMed, the Cochrane Database of Systematic Reviews, Web of Science Core Collection, and Google Scholar. Search terms combined concepts related to physical activity and exercise with cancer, fascia, autonomic function, and related mechanistic themes to capture clinical, translational, and preclinical evidence. Study selection was guided by relevance to the review aim and methodological rigor, with preference given to randomized controlled trials, systematic reviews and meta-analyses, large prospective cohort studies, mechanistic preclinical investigations, and clinical guidelines from recognized professional organizations. Reference lists of key articles and relevant review papers were manually screened to identify additional eligible studies. Non-peer-reviewed sources, studies without empirical data, and intervention studies with fewer than 50 participants were excluded. In total, approximately 80 primary research articles, systematic reviews, and meta-analyses were synthesized. To ensure methodological transparency, a detailed breakdown of all included studies - categorized by study type (e.g., RCTs, prospective cohorts, animal models, *in vitro* studies), population characteristics, primary outcomes, main findings, and Digital Object Identifiers (DOIs) - is provided in the Supplementary Materials (**Supplementary Table 1**).

### 2.2 Evidence quality assessment

Preliminary literature mapping was conducted using structured keyword searches, and all candidate references were subsequently retrieved, reviewed in full, and manually verified against primary sources prior to inclusion. Evidence quality and certainty were qualitatively appraised using established methodological frameworks as reference guides - specifically, the Cochrane Risk of Bias tool for randomized controlled trials, AMSTAR-2 for systematic reviews, and the Newcastle-Ottawa Scale for prospective cohort studies. Given the narrative rather than systematic design of this review, these frameworks were utilized exclusively to inform qualitative judgments regarding evidence strength and to guide the GRADE-based characterization of overall certainty, rather than serving as quantitative scoring instruments or formal meta-analytic pooling mechanisms.

This approach is consistent with established practice for narrative reviews that seek to synthesize mechanistic and clinical evidence across heterogeneous study designs (2, 3).

## 3 Exercise mechanisms in cancer biology

### 3.1 Tumor microenvironment normalization

The tumor microenvironment (TME) is a complex ecosystem comprising cancer cells, infiltrating immune cells, cancer-associated fibroblasts (CAFs), endothelial cells, and extracellular matrix (ECM) components that collectively establish a hostile landscape for anti-tumor immunity. Abnormal vasculature, characterized by tortuous, leaky vessels, generates regions of hypoxia, acidosis, and elevated interstitial fluid pressure (IFP), conditions that promote treatment resistance and metastatic potential (8).

Regular aerobic exercise increases circulating endothelial progenitor cells and modulates VEGF signaling, promoting vascular maturation and pericyte coverage of tumor vessels in preclinical breast cancer models (9). However, direct human evidence supporting exercise-induced tumor vascular normalization remains scarce, and meta-analytic data suggest that regular exercise interventions exert highly limited and inconsistent effects on circulating angiogenesis-related biomarkers, including VEGF, in cancer patients (10). Thus, the pronounced vascular normalization effects observed in animal models must not be assumed to occur uniformly in clinical populations.

In pancreatic ductal adenocarcinoma models, exercise-induced endothelial shear stress activates calcineurin–NFAT–TSP1 signaling, yielding structurally more functional vessels with reduced leakage, lower IFP, and a 24% increase in lectin-positive perfused vessels (11). These morphological changes have been associated with improved tumor oxygenation and enhanced chemotherapy delivery in preclinical models; direct human validation of effect magnitudes remains an active area of investigation (9, 11).

Hypoxia reduction and immunosuppression reversal: Reduction in intratumoral hypoxia carries substantial therapeutic implications. Hypoxia drives glycolytic metabolism in cancer cells, generating lactate that acidifies the TME, impairs effector immune cell function, and promotes immunosuppressive regulatory T cell (Treg) differentiation at the expense of effector T cells (12). HIF-1α signaling directly induces expression of programmed death ligand-1 (PD-L1), a primary mechanism of resistance to immune checkpoint inhibitor therapy (13).

Preclinical studies demonstrate that exercise reduces tumor hypoxic area and attenuates HIF-1α-associated biomarker expression, though the reported effect magnitudes vary across diverse tumor models, and context-dependent expression profiles have been documented (12, 14, 15). Consequently, care must be taken when extrapolating these specific animal-model outcomes across heterogeneous cancer types.

By normalizing vascular perfusion and reducing hypoxia-driven immunosuppression, exercise enhances immunotherapy responsiveness - an effect supported by preclinical chemotherapy sensitization data and emerging translational evidence (12, 14, 15).

### 3.2 Anti-tumor immunity enhancement

Acute exercise reliably boosts circulating anti-tumor effectors like NK and T cells (16), but chronic training shows inconsistent systemic effects, contrasting with Neuro-Immuno-Fascial (NIF) mechanisms that target fascial remodeling for sustained TME modulation (13). While exercise induces transient sympathomimetic shifts, NIF leverages mechano-neural pathways to reduce fibrosis and enhance infiltration of anti-tumor immune effector cells (such as NK cells and CD8^+^ T lymphocytes), offering complementary potential of fascial manipulation (16, 17).

Exercise training may enhance specific NK functions in subsets of studies, such as increased NKp46 expression (activating receptor) on CD56dim NK cells or preserved degranulation during chemotherapy, though overall NKCA remains unchanged. Regular exercise promotes anti-inflammatory shifts, reduces chronic cortisol/catecholamine elevations (normalizing diurnal rhythms), and supports NK survival/production indirectly via lowered bone marrow suppression. Preclinical data suggest exercise boosts NK tumor infiltration, warranting future RCTs standardizing absolute NK counts, functional markers (e.g., NKp46, CD107a), and tumor microenvironment assessments (16, 18–22).

### 3.3 Circulating tumor cell reduction

Exercise acutely mobilizes immune cells and imposes hemodynamic shear stress on CTCs, while chronic training has been associated with reduced CTC burden in cancer patients. A mechanistically distinct pathway involves the physical destruction of CTCs through exercise-induced hemodynamic forces: *in vitro* exposure to approximately 60 dynes/cm² shear stress (in maximal aerobic exercise conditions) causes acute necrosis and subsequent apoptosis across multiple CTC lines, with comparatively less damage to normal blood cells (23). Notably, some CTC subpopulations may develop adaptive mechanoresistance through cytoskeletal remodeling, underscoring the complexity of this pathway and the need for caution in extrapolating microfluidic findings directly to human physiology.

Human studies offer partial clinical corroboration. Breast cancer survivors engaging in structured aerobic exercise at both low-dose (150 min/week) and high-dose (300 min/week) demonstrated significant reductions in CTCs compared to non-exercising controls, with a mean reduction of approximately 45% (24). The dose-dependent relationship between exercise intensity and CTC reduction is consistent with a hemodynamic shear stress mechanism, though concurrent exercise-induced NK cell mobilization and myokine secretion cannot be excluded as contributing pathways (24).

### 3.4 Systemic metabolic-inflammatory reprogramming

Exercise training in cancer survivors reprograms systemic metabolism and inflammation, producing adaptations that counteract tumor-promoting mechanisms. Regular aerobic and resistance exercise - particularly at moderate-to-high intensity - enhances insulin sensitivity (25, 26). These improvements reduce circulating insulin and IGF-1, two potent mitogenic factors that drive tumor growth through the PI3K/AKT pathway (27, 28).

Meta-analyses of ≥8-week exercise interventions consistently report significant serum IGF-1 reductions of about –14.7 ng/mL in survivors, contrasting with the increases seen in healthy or obese populations (28, 29). In parallel, IGF-binding protein-3 (IGFBP-3) rises, limiting IGF-1 bioavailability and further diminishing mitogenic signaling (30). Mechanistic explanations include exercise-induced caloric deficit, improved hepatic regulation, and enhanced skeletal muscle glucose uptake. These systemic adjustments are linked to lower cancer recurrence risk and extended survival (31, 32).

Epidemiological evidence supports this mechanistic link: elevated IGF-1 is associated with greater recurrence and mortality risk in breast and prostate cancer (HR 1.25–1.31), particularly in survivors with metabolic syndrome (33–35). Conversely, sustained physical activity reduces recurrence by 20–30% and mortality by 30–40% in breast cancer survivors, with the largest gains observed in those maintaining ≥150 min/week of exercise (26).

While some structured programs have shown reductions in pro-inflammatory cytokines such as TNF-α or IL-6, recent meta-analyses demonstrate that the effects of exercise on anti-inflammatory biomarkers, including IL-10 and adiponectin (32), are generally small, inconsistent, or non-significant in cancer survivors (26). Furthermore, these biomarker alterations appear more prominent as transient, acute exercise responses rather than sustained, long-term training adaptations (26). These systemic shifts help restore metabolic homeostasis and suppress chronic inflammation, creating a less favorable environment for tumor progression.

These metabolic-anti-inflammatory shifts prime the immune system for enhanced anti-tumor responses, particularly when exercise synergizes with modern immunotherapies, as preclinical models demonstrate.

### 3.5 Exercise-immunotherapy synergy

Immune checkpoint inhibitors (ICIs) such as anti-PD-1, anti-PD-L1, and anti-CTLA-4 depend partly on adequate T-cell abundance, activation, and tumor infiltration to achieve meaningful clinical effects (36). In preclinical models of immunologically “cold” tumors, the addition of structured exercise to anti-PD-1 therapy has been associated with slower tumor progression, greater cytotoxic T-cell infiltration, and more pronounced tumor cell apoptosis (37). These studies further suggest that exercise may modulate T-cell exhaustion phenotypes and, via IL-15–related pathways, support the maintenance of central memory T-cell populations, thereby creating a more permissive immune context for ICI activity (37–44). Taken together, these findings indicate a biologically plausible interaction between exercise and ICIs in animal models, but confirmation of comparable synergistic effects in human patients receiving immunotherapy remains limited and requires carefully designed clinical trials (36, 37).

## 4 Fascia-autonomic-TME axis

### 4.1 Fascial architecture and cancer: an overview

Fascia is increasingly recognized as a biologically active connective tissue network that integrates mechanical, neural, vascular, and immune signals across tissue compartments (7, 45). Histological and immunohistochemical studies indicate that fascial tissue is richly innervated and vascularized, with autonomic fibers comprising a substantial proportion of its neural supply, supporting the view that fascia may function as a responsive mechano-sensory interface rather than as a purely passive scaffold (45). In oncology, this structural continuity is relevant because fascial extracellular matrix is connected with peri-tumoral stroma, where altered tissue mechanics, stromal remodeling, and hypoxia contribute to tumor progression and immune exclusion (7, 14, 37).

Physiological mechanical loading appears capable of influencing fascial biology, whereas pathological or sustained adverse remodeling may contribute to fibrosis, altered matrix organization, and tumor-permissive stromal conditions (7). Accordingly, appropriate mechanical loading may promote adaptive collagen remodeling and improved tissue compliance, with possible downstream relevance for the tumor microenvironment (7). However, the extent to which these biomechanical adaptations translate into meaningful anti-tumor effects in human oncology remains uncertain, since much of the mechanistic evidence remains preclinical or conceptual (7, 14, 37).

### 4.2 Structural and functional organization of fascia in tumor microenvironment regulation

The fascial system forms a continuous body-wide network with mechanotransductive properties that may influence interstitial pressure, tissue hydration, vascular perfusion, and local cellular signaling (7). This structural continuity supports the concept that fascia may participate in the regulation of stromal mechanics rather than functioning solely as passive connective tissue (7, 45). Within the tumor stroma, this framework is hypothesized to interact with cancer-associated fibroblast-driven extracellular matrix remodeling, although direct demonstration of these processes in exercising oncology patients is currently lacking (7, 45).

**Figure 2:**
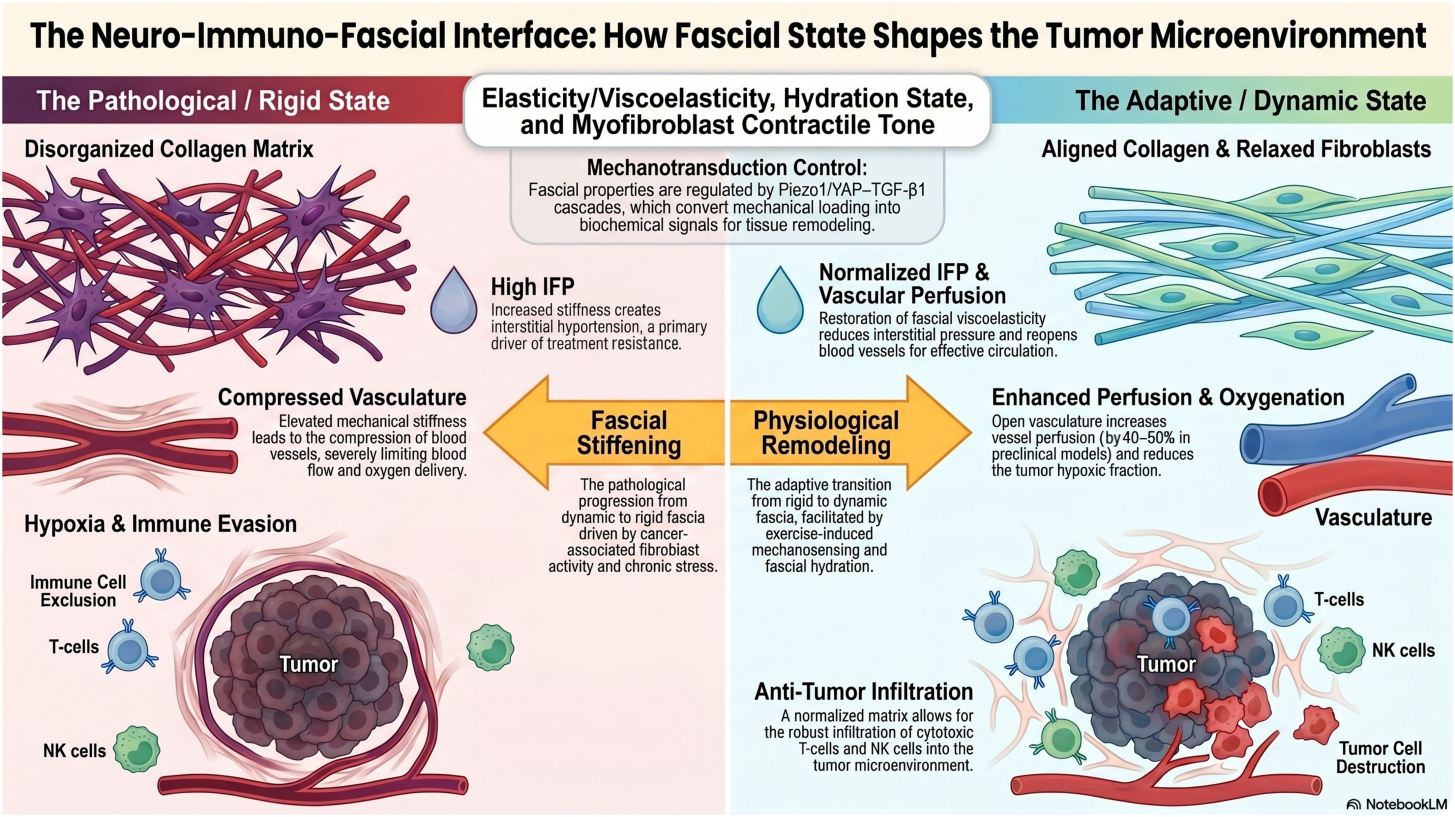
Mechanobiological regulation of the tumor microenvironment via the fascial interface (conceptual model). Schematic comparison of two stylized fascial–stromal states surrounding a tumor. The left panel depicts a pathological configuration characterized by disorganized, densely crosslinked collagen, elevated myofibroblast tone, increased interstitial fluid pressure, vascular compression, hypoxia, and reduced immune-cell access, features that are associated with an “immunologically cold” tumor microenvironment in preclinical and translational studies. The right panel illustrates an adaptive, mechanically more compliant fascial architecture that may arise under appropriate physiological loading (e.g., exercise or selected physical therapies), with improved tissue mobility, more favorable interstitial and vascular dynamics, and conditions that could support better perfusion and immune-cell trafficking. This figure is intended as a hypothesis−generating framework based on mechanobiological and preclinical data. It does not imply that such fascial states or transitions have been directly quantified or causally linked to anti−tumor effects in human oncology cohorts.

The biophysical properties of fascia, including elasticity, viscoelasticity, hydration state, and myofibroblast tone, may affect interstitial fluid dynamics and vascular compression, thereby contributing to stromal conditions associated with hypoxia and immune exclusion (7, 14). **Figure 2** schematically contrasts these two fascial states, illustrating how a pathological, rigid matrix with disorganized collagen and elevated myofibroblast tone compresses vasculature and excludes immune cells, versus an adaptive, dynamic matrix with aligned collagen and relaxed fibroblasts that supports normalized interstitial pressure, enhanced perfusion, and immune-cell infiltration. Excessive matrix stiffening and elevated interstitial pressure are mechanistically relevant because they may impair perfusion and reinforce tumor-permissive microenvironments (7, 14). Nevertheless, targeting fascial biomechanics should at present be regarded as a biologically plausible research direction rather than an established oncologic therapeutic strategy (7).

### 4.3 Cancer-associated fibroblasts, ECM remodeling, and the psycho-fascial stress axis

Beyond its structural role, fascia may participate in bidirectional interactions between psycho-emotional stress, autonomic regulation, and stromal remodeling (7). Chronic stress states can activate the hypothalamic–pituitary–adrenal axis and sympathetic signaling, with downstream effects on myofibroblast activity, cytokine release, and extracellular matrix densification (7). Together, these processes may contribute to tissue conditions that favor inflammation, stiffness, and impaired immune surveillance, although the magnitude and clinical relevance of these pathways in cancer patients remain incompletely defined (7).

This proposed psycho-fascial axis may also operate in a feedback manner, whereby fascial densification amplifies nociceptive input and sustains autonomic dysregulation (7, 45). Targeted fascial interventions may help address symptom burden and psycho-physiological dysregulation; whether they alter tumor-permissive stromal mechanics in humans remains unknown (7). For this reason, such interventions are best framed as supportive components of integrative care, complementing exercise oncology and other standard therapies, rather than as tumor-directed treatments (7, 50).

### 4.4 Fascia, ANS dysregulation, and tumor microenvironment signaling

Cancer and cancer-related treatments are frequently associated with autonomic imbalance, characterized by reduced vagal activity and heightened sympathetic tone, both of which have been linked to inflammatory and pro-tumorigenic signaling (7, 50). Because fascia is densely innervated and mechanically responsive, it represents a plausible interface through which autonomic, mechanical, and stromal processes could interact (7, 45). In this context, fascial stiffening, altered tissue glide, and peri-neural mechanical stress may amplify autonomic dysregulation, while sympathetic activation may further reinforce stromal remodeling and tumor-supportive conditions (7).

Exercise may influence this proposed fascia–ANS–tumor microenvironment axis through improvements in autonomic regulation and tissue mobility (7, 50). Meta-analytic evidence supports exercise-related improvements in heart rate variability in cancer survivors, suggesting better autonomic balance, while mechanobiological models propose that exercise-induced tissue loading could affect fascial hydration, viscoelastic behavior, and downstream signaling (7, 50). However, direct evidence that these changes alter tumor stromal mechanics, immune-cell trafficking, or oncologic outcomes in humans remains limited, and this relationship should therefore be interpreted as a hypothesis-generating framework rather than a validated clinical mechanism (7, 14, 37, 50).

### 4.5 Fascial remodeling as a therapeutic target

The fascial system is increasingly discussed as a potential mechanistic link connecting exercise−induced changes in autonomic balance, systemic inflammation, vascular perfusion, and tumor microenvironment adaptation, but remains an exploratory concept rather than an established therapeutic target.

By reconceptualizing fascia as an active neuro-immuno-mechanical interface rather than a passive scaffold, exercise oncology can be understood as functioning through integrated remodeling of fascial architecture, ANS balance, and local TME signaling. This perspective aligns emerging fascia research with established exercise oncology mechanisms and identifies new therapeutic targets for optimizing exercise interventions in cancer care (51–65).

### 4.6 Clinical and therapeutic applications of the NIF axis

#### 4.6.1 Autonomic nervous system dysregulation in cancer

Meta-analyses and systematic reviews support that exercise interventions improve heart rate variability (HRV) in cancer patients and survivors. These changes indicate better ANS balance, countering the reduced parasympathetic activity often seen post-cancer therapy (50, 66) (**Table 1**).

**Table 1:** Effect of exercise type on Heart rate variability (HRV).

Exercise type	Key HRV improvements	Evidence level	Notes [sources]
Aerobic + Resistance (CART/Circuit)	↑ SDNN (MD 12.79 ms), ↑ RMSSD (MD 13.08 ms), ↑ HF ↓LF/HF ratio (MD −0.32) (50)	High (meta-analysis of 6 RCTs) (50, 66)	Moderate intensity; best for overall autonomic balance
Resistance Alone	↑ HF power	Moderate (68) ​	Boosts parasympathetic; less data in cancer
Mind-Body (Qigong/Tai Chi)	↑ TP, variable SDNN/RMSSD	Low ​ (50)	Lighter intensity; smaller effects ​ (68)
High-Intensity Interval (HIIT)	↑ SDNN/RMSSD (general adults)	Emerging ​ (68)	Limited cancer-specific data; promising for fitness

CART, combined aerobic and resistance training; SDNN, standard deviation of normal-to-normal intervals; MD, mean difference; RMSSD, root mean square of successive differences; HF, high-frequency power; LF, low frequency power; TP, total power; RCTs, randomized controlled trials. LF/HF ratio interpretation reflects mixed sympathetic-parasympathetic modulation and should be interpreted cautiously (50).

The '↑' symbol indicates a statistically significant increase in the corresponding heart rate variability (HRV) parameter.

The ↓ symbol indicates a statistically significant decrease in the corresponding HRV parameter.

A 2021 systematic review, a meta-analysis of six RCTs (272 participants, aged 30–75), and a review of eight trials confirmed that exercise significantly increased HRV parameters, thereby being beneficial for cancer treatment (50, 66, 67).

#### 4.6.2 Heart rate variability as an autonomic biomarker in oncology

For oncology practice, structured moderate-intensity exercise (e.g., aerobic + resistance) during/after treatment enhances autonomic recovery, potentially aiding survivorship (50, 66).

Aerobic and resistance training (individually or combined) show the strongest evidence for improving HRV metrics like standard deviation of normal-to-normal intervals (SDNN) and root mean square of successive differences (RMSSD) in cancer survivors, based on meta-analyses and RCTs. Mind-body exercises like Qigong or Tai Chi yield smaller or inconsistent gains, possibly due to lower intensity (50, 67) (**Table 1**).

Programs typically last 8–16 weeks, 3 sessions/week, 60–90 min each, at moderate intensity (e.g., 35-85% heart rate reserve). Aerobic components (walking, cycling, elliptical) paired with resistance (bands, weights, bodyweight squats/presses) or circuit training boost parasympathetic markers most reliably (50, 66, 67).

Higher intensities may enhance gains via catecholamine release and vagal stimulation but start moderate for safety (50, 66). Despite these findings, larger trials are needed to optimize protocols.

### 4.7 Clinical evidence: outcomes and survivorship

The mechanistic pathways described in Sections 3 and 4, encompassing vascular normalization, immune enhancement, metabolic reprogramming, and fascial-autonomic remodeling, serve as conceptual frameworks to explore how physical activity might interact with tumor biology. Clinically large-scale prospective cohort analyses demonstrate dose-dependent reductions in all-cause cancer mortality of 20–47% across tumor entities, with the greatest benefit observed in survivors maintaining ≥150 minutes per week of moderate-to-vigorous physical activity (**Table 2**) (46, 47).

**Table 2:** Post-diagnosis physical activity and mortality risk reduction in cancer survivors.

Outcome	Highest vs. lowest PA (HR, 95% CI)	Optimal dose	Population characteristics
All-Cause Mortality	0.63 (0.56-0.71)	≥10 MET-h/week	Median age 62–68 years; follow-up >9 years (46, 47)
Cancer-Specific Mortality	0.70 (0.60-0.82)	150–360 min/week moderate-vigorous	Breast/colorectal predominant; n>20,000 (48, 49)

PA, Physical Activity; HR, Hazard Ratio; CI, Confidence Interval; MET, Metabolic Equivalent of Task; MET/h, MET-hours per week.

The landmark CCTG CO.21 CHALLENGE randomized controlled trial, the first exercise RCT powered to detect survival endpoints, established that a structured three-year supervised exercise program initiated after adjuvant chemotherapy reduced disease recurrence and new primary tumors by 28% and all-cause mortality by 37% in stage II–III colon cancer survivors over a median follow-up of 7.9–8.9 years (6). This represents an effect magnitude comparable to some adjuvant chemotherapy regimens within this specific population (6).

Similarly, specific physiological metrics often highlighted in unified framework diagrams, such as a 30–45% reduction in tumor hypoxia or a 40–60% improvement in vessel perfusion, are observed strictly within highly controlled preclinical animal models and specific experimental configurations. They do not represent established average effect magnitudes within human clinical populations, where systemic and tissue-level adaptations remain highly variable.

## 5 Safety, implementation, and exercise prescription

Translating the clinical and mechanistic evidence reviewed herein into practice requires attention to safety screening, individualized prescription, and implementation frameworks appropriate to the heterogeneity of cancer patients across disease stages and treatment phases. Current international guidelines from the American College of Sports Medicine (ACSM) and the Clinical Oncology Society of Australia (COSA) recommend that cancer patients avoid inactivity and engage in at least 150 minutes per week of moderate-intensity aerobic exercise combined with two or more resistance training sessions per week, adapted for treatment-related toxicities including anemia, neuropathy, bone metastases, and immunosuppression (3, 69). Pre-exercise screening using validated tools, including the Physical Activity Readiness Questionnaire for Everyone (PAR-Q+) and oncology-specific adaptations, is recommended to stratify risk and identify contraindications, with particular vigilance for cardiotoxicity risk in patients receiving anthracyclines or trastuzumab (2).

Emerging precision exercise oncology approaches advocate for individualized dosing algorithms integrating tumor type, treatment phase, and autonomic/structural status. However, it must be explicitly stated that advanced assessment tools, such as HRV spectral analysis and shear-wave elastography, remain strictly investigational within oncology settings. These modalities currently lack validation as clinically actionable or predictive biomarkers and require rigorous testing in prospective trials before integration into routine clinical practice (17, 66, 70).

## 6 Limitations

Several limitations of this review warrant acknowledgment. First, as a narrative rather than systematic review, the synthesis is inherently subject to selection bias in the choice of included studies; although structured database searches (PubMed, Cochrane, Web of Science) were conducted and evidence quality was assessed using GRADE, AMSTAR-2, and Newcastle-Ottawa Scale criteria. The absence of a pre-registered protocol and PRISMA-compliant flow diagram limits reproducibility and precludes formal meta-analytic pooling (2, 3). Second, the mechanistic evidence underpinning the NIF framework, particularly Piezo1/YAP–TGF-β1 mechanotransduction, fascial ECM remodeling, and exercise-induced CAF phenotype modulation, derives predominantly from preclinical *in vitro* and murine models. CAF-mediated ECM stiffening attenuation through mechanical loading has not been directly validated in exercising cancer patients. Furthermore, the translational gap between mechanobiological findings and measurable fascial outcomes represents a critical area for future clinical investigation (7, 39, 45, 70). Third, the exercise oncology trials included in this review are highly heterogeneous with respect to exercise modality, intensity, duration, cancer type, treatment phase, and patient population, constraining the generalizability of effect size estimates and comparison across studies (71–73). Fourth, validated clinical tools for fascial phenotyping, including shear-wave elastography and HRV spectral analysis as measures of autonomic-fascial status, have not yet been validated as biomarkers in oncology settings, limiting the clinical applicability of the precision exercise oncology framework proposed here (12, 72). Fifth, available clinical data on tumor-stromal physical interactions are predominantly restricted to solid tumor models. The clinical applicability of the NIF framework remains uncharacterized in malignancies with fundamentally different structural profiles, such as hematological or non-solid cancers with distinct architectural frameworks (73–75). These limitations notwithstanding, the convergent mechanistic and clinical evidence reviewed herein supports the biological plausibility of the NIF interface as an integrative framework and identifies clear priorities for prospective trial design and biomarker development.

## 7 Conclusion and future perspectives

The converging evidence synthesized in this review positions the neuro-immuno-fascial (NIF) interface as a biologically coherent, hypothesis-generating concept that may help integrate vascular, immunological, autonomic, and mechanobiological dimensions of exercise oncology. Established clinical evidence supports structured physical activity as part of standard cancer care in selected settings, whereas the specific fascial-stromal and autonomic mechanisms proposed within the NIF model remain incompletely characterized and largely unvalidated in humans.

Future research should focus on three translational priorities: first, prospective trials that examine how different exercise modalities interact with tumor biology and standard oncologic therapies; second, validation of candidate biomarkers such as heart rate variability and shear-wave elastography in oncology populations; and third, cautious development of individualized exercise frameworks only after these measures demonstrate reproducibility, prognostic value, and clinical utility.

Overall, the NIF interface offers a useful conceptual structure for future mechanistic and translational research, but it should not currently be interpreted as an established clinical pathway or decision-making tool.

## Glossary

ANS: Autonomic Nervous System
ACSM: American College of Sports Medicine
AMSTAR-2: A Measurement Tool to Assess systematic Reviews (version 2)
BDNF: Brain-Derived Neurotrophic Factor
CAF: Cancer-Associated Fibroblast
CART: Combined Aerobic and Resistance Training
CCTG: Canadian Cancer Trials Group
CI: Confidence Interval
CIPN: Chemotherapy-Induced Peripheral Neuropathy
COSA: Clinical Oncology Society of Australia
CTC: Circulating Tumor Cell
CTLA-4: Cytotoxic T-Lymphocyte-Associated Protein 4
ECM: Extracellular Matrix
EPC: Endothelial Progenitor Cell
FAK: Focal Adhesion Kinase
FRS: Fascia Research Society
GRADE: Grading of Recommendations Assessment, Development and Evaluation
HF: High-Frequency (power, HRV domain)
HIF-1α: Hypoxia-Inducible Factor-1 alpha
HPA: Hypothalamic-Pituitary-Adrenal (axis)
HR: Hazard Ratio
HRV: Heart Rate Variability
ICI: Immune Checkpoint Inhibitor
IFP: Interstitial Fluid Pressure
IGF-1: Insulin-Like Growth Factor 1
IGFBP-3: Insulin-Like Growth Factor Binding Protein 3
IL-1β: Interleukin-1 beta
IL-6: Interleukin-6
IL-8: Interleukin-8
IL-10: Interleukin-10
IL-15: Interleukin-15
LF: Low-Frequency (power, HRV domain)
LPS: Lipopolysaccharide
MD: Mean Difference
MET: Metabolic Equivalent of Task
MET/h: MET-hours per week
NFAT: Nuclear Factor of Activated T cells
NIF: Neuro-Immuno-Fascial (interface)
NK: Natural Killer (cell)
NKCA: Natural Killer Cell Activity
PA: Physical Activity
PAR-Q+: Physical Activity Readiness Questionnaire for Everyone
PD-1: Programmed Cell Death Protein 1
PD-L1: Programmed Death Ligand 1
PI3K/AKT: Phosphoinositide 3-Kinase/Protein Kinase B (pathway)
RCT: Randomized Controlled Trial
RMSSD: Root Mean Square of Successive Differences
SDNN: Standard Deviation of Normal-to-Normal intervals
SPARC: Secreted Protein Acidic and Rich in Cysteine
TGF-β/TGF-β1: Transforming Growth Factor beta/beta-1
TH: Tyrosine Hydroxylase
TME: Tumor Microenvironment
TNF-α: Tumor Necrosis Factor alpha
TP: Total Power (HRV domain)
Treg: Regulatory T Cell
TSP1: Thrombospondin-1
VEGF: Vascular Endothelial Growth Factor
YAP: Yes-Associated Protein
