# Supplementary material for: Physical activity and the Neuro-Immuno-Fascial interface in cancer patients: mechanisms, clinical evidence, and therapeutic integration
Source: Front Oncol. 2026 Jul 31;16:1894580. doi: 10.3389/fonc.2026.1894580 (PMC13474146; doi:10.3389/fonc.2026.1894580)
Supplement: Supplementary file 1 [file Table1.docx]

**Supplementary Table S1: Evidence Matrix of Included Studies**

| **Citation / DOI** | **Study Type** | **Population / Model** | **Primary Outcomes Assessed** | **Main Findings & Certainty Level** |
| --- | --- | --- | --- | --- |
| **Courneya et al., 2025**    *(CCTG CO.21 CHALLENGE)*    [doi: 10.1056/NEJMoa250276] | Phase III RCT | n=889 stage II–III colon cancer survivors | Disease-Free Survival (DFS), Overall Survival (OS), recurrence rates | 3-year structured exercise reduced disease recurrence by 28% (HR = 0.72) and mortality by 37%. Certainty: High for this specific cohort; cannot be generalized to all cancer types. |
| **Lavín-Pérez et al., 2021**      [doi: 10.3389/fpsyg.2021.712823] | Systematic Review & Meta-analysis | 6 RCTs (n=272 cancer patients/survivors, ages 30–75) | Heart Rate Variability (HRV) parameters (SDNN, RMSSD) | Exercise significantly increased HRV parameters (MD SDNN: 12.79 ms; MD RMSSD: 13.08 ms). Combined aerobic and resistance training (CART) demonstrated the most robust improvements. Certainty**:** High. |
| **Wang et al., 2025**      [doi: 10.3389/fimmu.2025.1663560] | Systematic Review & Meta-regression | Diverse cancer patient cohorts | Systemic metabolic and inflammatory biomarkers (TNF-α, IL-6, IL-10, adiponectin) | Documented reductions in pro-inflammatory markers (TNF-α, IL-6). Notably, changes in anti-inflammatory markers like IL-10 and adiponectin were found to be small, inconsistent, or non-significant. Certainty: Moderate. |
| **Regmi et al., 2017**      [doi: 10.1038/s41598-017-03997-5] | Mechanistic *In Vitro* Study | Multiple Circulating Tumor Cell (CTC) lines | Cell necrosis and apoptosis under mechanical fluid forces | Exposure to 60 dyn/cm^2^ fluid shear stress (simulating maximal aerobic exercise conditions) causes direct mechanical destruction and apoptosis of CTCs. Certainty: High (*In Vitro* mechanics only). |
| **Hou et al., 2026**      [doi: 10.3389/fspor.2026.1774978] | Preclinical Animal Model | Pancreatic ductal adenocarcinoma (PDAC) murine models | Endothelial shear stress, calcineurin–NFAT–TSP1 signaling, vessel perfusion | Aerobic exercise-induced shear stress activates calcineurin pathways, yielding functional vessel remodeling, reduced leakage, and a 24% increase in lectin-positive perfused vessels. Certainty: Moderate (Translational gap to humans remains). |
| **Schadler et al., 2016**      [doi: 10.18632/oncotarget.11662] | Preclinical Animal Model | Murine tumor models | Tumor vascular normalization, vessel perfusion, chemotherapy efficacy | Aerobic exercise drives tumor vascular normalization, increasing vessel perfusion by 40–60% and reducing tumor hypoxic fraction by 30–45%, which synergistically enhances chemotherapeutic efficacy. Certainty: High in rodents; speculative/unvalidated in humans. |
| **Yan et al., 2023**      [doi: 10.3389/fimmu.2023.1265914] | Preclinical Animal Model | B16 murine melanoma cell models | Hypoxia modulation, T-cell infiltration, anti-PD-1 immunotherapy sensitivity | Voluntary exercise acts as a hypoxia modulator, counteracting T-cell exhaustion and robustly enhancing cytotoxic T-lymphocyte tumor infiltration when combined with anti-PD-1 therapy. Certainty: High in rodent models. |
| **Kwon et al., 2025**      [doi: 10.1080/07853890.2025.2586331] | Systematic Review & Meta-analysis | Obese, healthy, and cancer populations | Circulating Insulin-like Growth Factor 1 (IGF-1) | Interventions of ≥8 weeks consistently report significant serum IGF-1 reductions (mean: -14.7 ng/mL) in cancer survivors, reducing mitogenic signaling via the PI3K/AKT pathway. Certainty: High. |
| **Fede et al., 2021**      [doi: 10.3390/ijms22031411] | Histological / Quantitative Immunohistochemistry | Human deep/muscular fascial tissue samples | Innervation tracking, S100/Tyrosine Hydroxylase (TH) positivity ratios | Demonstrated that fascial tissue is highly vascularized and innervated. Quantitative ratios (≈2.96) estimate that roughly 33.82% of deep fascial nerve fibers are autonomic, facilitating neuro-mechanical tissue crosstalk. Certainty: High. |
